# A Murine Model to Study the Antibacterial Effect of Copper on Infectivity of *Salmonella Enterica* Serovar Typhimurium

**DOI:** 10.3390/ijerph8010021

**Published:** 2010-12-24

**Authors:** Riti Sharan, Sanjay Chhibber, Robert H. Reed

**Affiliations:** 1 Centre for Plant and Water Science, Faculty of Sciences, Engineering and Health, CQ University, Rockhampton, Queensland 4702, Australia; E-Mail: r.reed@cqu.edu.au; 2 Department of Microbiology, Panjab University, Chandigarh 160014, India; E-Mail: sanjaychhibber8@sify.com

**Keywords:** copper, *Salmonella* Typhimurium, murine model, infectivity, phagocytosis, sub-lethal injury, ROS-neutralised, tissue damage

## Abstract

This study investigated the effect of copper as an antibacterial agent on the infectivity of *Salmonella enterica* serovar Typhimurium. Mice were infected orally with a standardized dose of unstressed *Salmonella* Typhimurium and copper-stressed cells of *Salmonella* Typhimurium. Bacterial counts in ileum, blood, liver and spleen were observed up to 168 h under normal aerobic conditions. Serum sensitivity, phagocytosis, malondialdehyde levels and histopathology were studied for both set of animals. A decreased bacterial count in the organs with mild symptoms of infection and a complete recovery by 48 h was observed in mice infected with copper-stressed bacteria. Histopathological examination of ileum tissue demonstrated regeneration of damaged tissue post-infection with copper-stressed bacteria and no malondialdehyde levels were detected after 24 h in ileum, spleen and liver. Exposure to copper sensitized *Salmonella* Typhimurium to the lytic action of serum and intracellular killing by peritoneal macrophages. It can be concluded that copper stress confers a decrease in the infectivity of healthy *Salmonella* Typhimurium in normal mice. This study highlights the significance of use of copper as an antibacterial agent against *Salmonella* Typhimurium in reducing the risk of incidence of *Salmonella* infections from contaminated water.

## 1. Introduction

Typhoid continues to be a concern in developing nations especially in South-East Asia, due to unsafe water, contaminated food and lack of basic sanitation measures across large numbers of the population [1]. *Salmonella enterica* serovar Typhi causes Typhoid fever in humans [2] with infants, children and adolescents being most strongly affected [3]. In addition, an annual estimate of 800,000 to 4,000,000 of non-typhoidal infections [4] with approximately 1.3 billion cases of *Salmonella* related human gastroenteritis are reported [5]. *Salmonella enterica* serovar Typhimurium is widespread in its environmental distribution in many parts of South-East Asia, as evidenced from its isolation from rivers, sewage and other waterbodies [6] and infections are mostly associated with consumption of water and food items that have been contaminated, often due to improper handling/storage [7].

The most common causes of human salmonellosis are reported to be serovars Typhimurium and Enteritidis accounting for 57–67% of total isolates annually [7]. *Salmonella* Typhimurium is the most common non-typhoidal serovar isolated in India [8] and has the potential to cause systemic infections in humans, leading to medical complications as it can survive in different reservoirs and is easily transmitted through water and poultry to humans [9]. Infection of mice with *Salmonella* Typhimurium causes a disease similar to the one caused by *Salmonella* Typhi in humans [10]. *Salmonella* Typhi though a major cause of morbidity and mortality in humans is avirulent in animals, including mice [11].

Storage of drinking water is a common practice in rural homes in developing nations such as India [12] and studies have demonstrated the spread of *Salmonella* Typhimurium through contamination of stored water [13]. A number of point-of-use disinfection methods have been used to combat such waterborne infectious bacterial diseases in stored water and these include boiling [14], biosand filters [15] and chlorination [16].

More recently, the antibacterial potential of water storage in copper and brass vessels against common waterborne pathogens such as *Escherichia coli*, *Enterococcus faecalis* [17–18], Salmonella and *Vibrio cholerae* [19] has been studied. Short-term storage of *E. coli* and *E. faecalis* for up to 48 h in a brass water storage vessel caused sub-lethal injury to the bacteria [17] as demonstrated by a higher bacterial count under enumeration conditions designed to neutralise the damaging effects of reactive oxygen species [20]. Equivalent studies on effect of solar disinfection on infectivity of *Salmonella* Typhimurium in mice demonstrated that bacterial cells exposed to irradiation for 1.5 h were less infective than their non-irradiated counterparts and did not pose a potential infection hazard [21].

The present study was conducted to observe the effect of storing water containing *Salmonella* Typhimurium in a copper water storage vessel when compared with *Salmonella* Typhimurium not exposed to such copper stress, by elucidating various host and bacterial mechanisms operative in pathogenesis of the bacterium.

## 2. Methods

### 2.1. Preparation of Bacterial Cultures and Copper Stressed Cells

*Salmonella* Typhimurium strain 1,251 was obtained from the Institute of Microbial Technology, Chandigarh. The strain was studied at Panjab University, Chandigarh, India. Stocks were maintained by sub-culturing every 15–20 days on nutrient agar (HiMedia, Mumbai, India). For experimental procedures, a single colony of the bacterial strain was inoculated into 100 mL of nutrient broth and incubated overnight at 37 °C without shaking. The overnight culture was then centrifuged at 5,300 g for 5 min at 5 °C and rinsed twice with 0.85% NaCl to remove all traces of growth medium and the count was adjusted to approximately 2.2 × 10^7^ CFU. To obtain copper-stressed bacteria, the pellet was suspended in the same volume of sterile distilled water and then diluted to 1:100, *i.e.*, 100 mL of bacterial suspension was added to 10 L of sterile distilled water, pH 7.0. This bacterial suspension was stored in the copper vessel for 6 h, the time of storage being standardized from preliminary experiments conducted in our laboratory wherein, it was observed that 6 h storage of water containing *E. coli* in a copper vessel resulted in an increased injured bacterial count without completely inactivating the cells as demonstrated by a higher count under reactive oxygen species neutralised conditions (ROS-neutralised) as compared with normal aerobic conditions [20]. The suspension was then centrifuged at 5,300 g for 5 min at 5 °C, rinsed twice with 0.85% NaCl and finally suspended in 0.85% NaCl and enumerated under normal aerobic conditions and under ROS-neutralised conditions. The final inoculum thus contained approximately 1.6 × 10^7^ CFU mL^−1^ of healthy uninjured bacterial cells and 4.7 × 10^7^ CFU mL^−1^ of injured bacterial cells (6.3 × 10^7^ CFU mL^−1^ ROS-neutralised count— 1.6 × 10^7^ CFU mL^−1^ healthy cell count).

### 2.2. Preparation of Copper and Control Water Storage Vessels

Sterile distilled water adjusted to pH 7.0 was used. Copper vessels (12 L capacity), obtained from the local market in Chandigarh were disinfected, scrubbed and rinsed thoroughly to remove any adherent contamination/dust from the inner surface. Glass flasks of equivalent volume were used as controls. The mouths of all vessels were kept covered with sterile paper during the test procedure, to prevent airborne contamination of the water stored within.

### 2.3. Experimental Animals

Outbred female LACA mice, 6−8 weeks old, weighing 20−25 g were used in the present study. The animals were obtained from The Central Animal House, Panjab University, Chandigarh, India. The present study was designed with an outbred strain of mice as the research required a vigorous supply of mice that could be obtained in reasonable numbers without large expense, and therefore specific considerations of genotypes were less important. All the animals were given an antibiotic-free diet and were provided with water *ad libitum*. For each experiment, two groups of 18 mice per group (2 mice per time point sample) were used; one group was administered unstressed *Salmonella* Typhimurium and the second group of mice was administered copper-stressed *Salmonella* Typhimurium. Each experiment was conducted in triplicate. All of the experiments were carried out strictly according to the guidelines and under the approval of the Animal Ethical Committee, Panjab University, Chandigarh.

### 2.4. Bacterial Load in Ileum, Blood, Liver and Spleen

Two groups of 18 mice each were deprived of food overnight. They were then fed with 100 μL of sodium bicarbonate, followed by infection using a gavage needle with (i) unstressed *Salmonella* Typhimurium and (ii) copper-stressed *Salmonella* Typhimurium. To determine bacterial loads in ileum, blood, liver and spleen, mice were sacrificed by rapid cervical dislocation at 0, 6, 24, 48, 72, 96, 120, 144 and 168 h post-infection. Organs were homogenized in 3 mL of 0.85% NaCl, serially diluted in 0.85% NaCl and then plated on nutrient agar plates. In case of blood counts, blood was extracted from the mice using cardiac puncture, appropriately diluted in 0.85% NaCl and plated onto nutrient agar plates and incubated under aerobic conditions.

### 2.5. Serum Bactericidal Test

The bactericidal action of serum against (i) unstressed *Salmonella* Typhimurium and (ii) copper-stressed *Salmonella* Typhimurium was tested according to the method of Taylor and Kroll (1983) [22]. 250 μL of either of the bacterial suspensions was incubated with 500 μL of pre-warmed pooled normal mice serum, then 10-fold dilutions were made in 0.85% NaCl at 0, 1, 2 and 3 h and 20 μL of each dilution was plated onto sterile, dry nutrient agar plates using the Miles and Misra surface droplet method [18]. The plates were then incubated overnight at 37 °C under aerobic conditions and the colony count was expressed as CFU mL^−1^ by adjusting for volume and dilution.

### 2.6. Phagocytosis

The phagocytic potential of peritoneal macrophages was assessed according to the method of Allen *et al.* (1987) [23]. Isolation of peritoneal macrophages was carried out using peritoneal lavage fluid of mice infected orally with unstressed as well as copper-stressed *Salmonella* Typhimurium. To obtain peritoneal lavage fluid, the mice peritoneal cavities were carefully exposed by surgically removing the skin layer of the abdomen using sterile scissors and forceps without disrupting the blood vessels. A volume of 8−10 mL RPMI-1640 medium was injected into the cavity and the abdomen was massaged for 1−2 min. The peritoneal lavage was sucked back using a syringe and added to a sterile glass Petri dish. The Petri dish was incubated at 37 °C for 1 h in a CO_2_ incubator to allow adherence of the macrophages to the surface of the dish. The supernatant was then removed and the layer of adherent cells was detached from the surface of glass using a rubber-covered glass rod. The macrophages were washed twice with RPMI-1640 after centrifuging at 102 g for 5 min.

Bacterial uptake by peritoneal macrophages was observed by adding 0.4 mL of mouse serum to 0.5 mL of a macrophage cell suspension (10^8^ cells mL^−1^) and 0.1 mL of (i) unstressed and (ii) copper-stressed bacterial suspension in separate sterile microcentrifuge tubes. The microcentrifuge tubes were then incubated at 37 °C under 5% CO_2_ atmosphere. After 0, 30, 60 and 90 min, a 20 μL aliquot of the cell suspension was withdrawn and added to 2 mL of ice cold RPMI-1640 medium, centrifuged at 330 g for 10 min and the bacterial count in the supernatant was determined by spreading 20 μL of serial 10-fold dilutions onto nutrient agar plates, incubated at 37 °C under aerobic conditions. The bacterial counts were expressed as the percentage of the initial inoculum taken up by peritoneal macrophages at the respective sampling times.

To determine intracellular inactivation of bacteria by peritoneal macrophages, bacterial opsonization was carried out by adding 4.5 mL of (i) unstressed and (ii) copper-stressed bacterial suspension to 0.5 mL of mouse serum and then incubating at 37 °C for 30 min under a 5% CO_2_ atmosphere. The cell suspensions were then washed twice with 2.5 mL of 0.85% NaCl. These opsonised bacterial suspensions were then added to 0.5 mL of macrophage cell suspension (10^8^ cells mL^−1^) in endotoxin-free RPMI 1640 medium-supplemented with 5% foetal calf serum containing no antibiotic. The suspensions were incubated for 20 min at 37 °C under a 5% CO_2_ atmosphere to allow bacterial inactivation to take place within peritoneal macrophages. Removal of non-phagocytosed bacteria was carried out by centrifuging the suspensions at 330 g for 5 min at room temperature and washing the pellets twice with 0.85% NaCl. The pellets thus obtained were suspended in 2 mL RPMI-1640 medium containing 5% foetal calf serum and 50 μg mL^−1^ of gentamycin and then incubated at 37 °C under a 5% CO_2_ atmosphere. The cells were lysed at 0, 1, 2, and 3 h post-incubation by suspending 1 mL aliquots in 0.85% NaCl containing 0.5% sodium deoxycholate. Subsequently, 20 μL aliquots of serial 10-fold dilutions of the cell suspensions were spread onto nutrient agar plates as before. Colony counts were carried out after overnight incubation at 37 °C under standard aerobic conditions and expressed as percentage of bacteria in peritoneal macrophages at each sampling time interval compared to the initial value.

### 2.7. Malondialdehyde (MDA) Estimation

Lipid peroxidation in ileal, spleen and liver tissue of two groups of mice infected orally with (i) unstressed and (ii) copper-stressed *Salmonella* Typhimurium was estimated according to the method of Wills (1965) [24]. Ileum, spleen and liver were excised from mice at 0, 6, 24, 48, 72, 96, 120, 144 and 168 h post-infection and homogenised in sterile homogenizing vials containing 3 mL of 0.85% NaCl. A volume of 0.2 mL of tissue homogenate was added to 0.045 mol H_2_SO_4_, 0.2 mL of 8.1% sodium dodecyl sulphate (SDS), 1.5 mL of 0.8% thiobarbituric acid and incubated at 100 °C for 1 h in a water bath. After cooling to room temperature, 1 mL of distilled water and 5 mL of butanol:pyridine (5:1) was added, centrifuged at 1,628 g for 10 min and the absorbance of the upper organic phase was measured at 532 nm using a Hitachi U2900 spectrophotometer. The results were expressed as nmol of MDA per mg protein. The protein estimation was carried out according to Lowry *et al*. (1951) [25].

### 2.8. Histopathological Examination

The ileum of two groups of mice infected orally with (i) unstressed (ii) and copper-stressed *Salmonella* Typhimurium was subjected to histopathological analysis. The ileum of experimental mice was excised at pre-determined time intervals post-infection and was fixed in 10% v/v formalin. Histological examination was then carried out by dehydrating, paraffin embedding, blocking, sectioning and staining with a standard hematoxylin eosin technique [26]. The sections were mounted in DPX.

### 2.9. Statistical Analysis

Sample means were statistically compared using student “*t*” tests, expressed in terms of probability (*p*).

## 3. Results and Discussion

Murine salmonellosis has long been studied as an alternative to human typhoid, in order to understand pathogenesis of the disease [27–28]. In the present study we have studied the effect of exposure to copper on infectivity of *Salmonella* Typhimurium by keeping the bacterium for a short-term in a copper water storage vessel, enumerating it under normal aerobic conditions to get the healthy bacterial count and under ROS-neutralised conditions to get the injured bacterial count and administering it orally into healthy mice. Our aim was to observe and understand if copper-stressed bacteria retain an ability to cause disease in a normal host. The finding of the present study has a significant practical value as it proves that storage of water contaminated with pathogenic *Salmonella* Typhimurium in a copper water storage vessel can decrease its pathogenesis in a mouse model. It also proves that injured bacteria are unable to cause disease in a normal host and is cleared by the host immune mechanism by 48 h. If the same applies to *Salmonella* Typhi, then this has a potential significance in relation to the incidence of waterborne diseases such as typhoid in rural areas where access to clean drinking water is an ongoing struggle.

**Figure 1:**
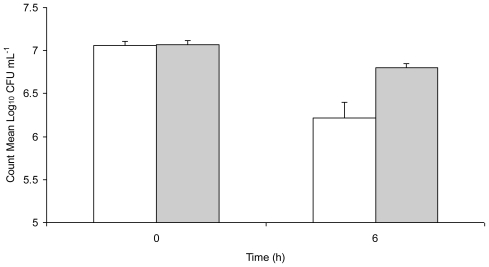
Plate counts of *Salmonella* Typhimurium 1251 in distilled water at pH 7.0 stored in a copper vessel at 30 °C and enumerated under (a) aerobic conditions (unshaded bars) and ROS-neutralised conditions (shaded bars). The dotted horizontal line represents minimum detection limit (log_10_ CFU mL^−1^ = 1.22). Error bars represent 95% confidence limits (*n* = 3).

Figure 1 shows inactivation and sub-lethal injury of *Salmonella* Typhimurium in water stored in a copper vessel for 6 h, enumerated under aerobic and ROS-neutralised conditions. There was a visible decrease in both aerobic and ROS-neutralised counts from 0 h to 6 h with a significant difference (*t* = 10.96, *p* = 0.008) between the two at 6 h. While the exact mechanisms of copper toxicity are unknown, and are probably not due to a single cause, the general biocidal properties of copper can be explained. Combination of reducing agents with copper ions causes an enhanced degradation of biological compounds by increasing oxidative damage [29]. In fact, the major toxic effect of copper has been proposed to be damage to lipids, nucleic acids and proteins by generation of toxic reactive oxygen species [30].

**Figure 2:**
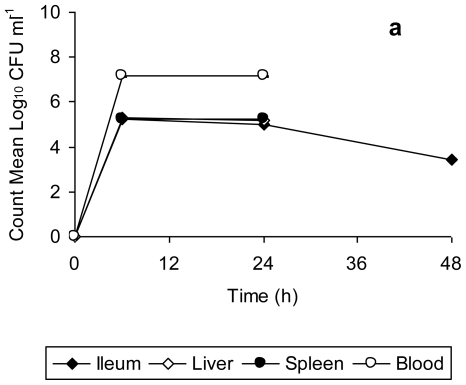

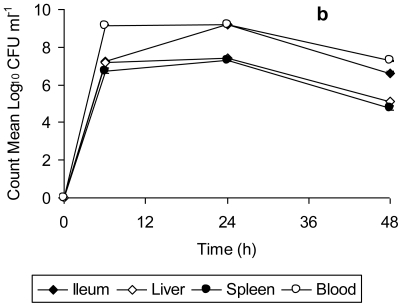
Bacterial count in ileum, spleen, liver and blood of mice orally infected with 2.2 × 10^7^ CFU of (a) unstressed *Salmonella* Typhimurium, (b) copper stressed *Salmonella* Typhimurium at 0, 6, 24, and 48 post infection. Error bars represent 95% confidence limits (*n* = 3). All the mice infected with unstressed *Salmonella* Typhimurium died after 48 h. All the mice infected with copper-stressed *Salmonella* Typhimurium survived with no counts obtained after 48 h from any of the organs.

Data for bacterial counts in the ileum, liver, spleen, and blood of mice infected with unstressed *Salmonella* Typhimurium (Figure 2a) and copper-stressed *Salmonella* Typhimurium (Figure 2b) is represented in Figure 2. The bacterial counts obtained from the organs and blood of mice infected with unstressed *Salmonella* Typhimurium increased from 6 h to 24 h post-infection, and decreased thereafter up to 48 h, this decrease being significant in the case of spleen (*t* = 63.08, *p* = 0.0002 from 24 h to 48 h). There was complete mortality of all test mice after 48 h, with the peak of infection at 24 h, as indicated by the bacterial load in organs (Figure 2a). In comparison, the bacterial counts in organs of mice infected with copper-stressed remained consistently slightly lower (Figure 2b). As compared to the experiments using unstressed cells, there was a slight decrease at 48 h in ileum and at 24 h in liver, spleen and blood. There was complete clearance of both healthy and injured bacteria from the ileum after 48 h and after 24 h in liver, spleen and blood, with no bacterial counts at any time point thereafter indicating the clearance of bacteria from these organs. The graphs represent time period up to 48 h only as no bacterial counts were obtained thereafter up to 168 h post-infection.

*Salmonella* Typhimurium is known to initiate infection by penetrating the intestinal epithelium of the small bowel, the area of initial localization being the terminal ileum [31]. Furthermore, a logarithmic increase in the bacterial count from gut associated lymphoid tissue (GALT) for 2 days post infection orally with *Salmonella* Typhimurium has been reported previously [32]. This is consistent with the findings of present study in which there was a progressive increase in the bacterial count in ileum of mice infected with unstressed *Salmonella* Typhimurium up to 48 h, followed by their death. In contrast, the clearance of bacteria from ileum of mice infected with copper-stressed *Salmonella* Typhimurium after 48 h is indicative of suppression/inhibition of the penetrating ability of both healthy and injured bacteria to penetrate into epithelium lining post-exposure to copper in the storage vessel. In natural *Salmonella* infections, bacteria gain entry into other organs such as liver and spleen by systemic circulation [33] with enlarged liver and swollen spleen visible by day 3 following infection with *Salmonella* Typhimurium [34]. In our study, we also observed a high bacterial count in blood, liver and spleen with slight swelling of the spleen of mice infected with *Salmonella* Typhimurium by 48 h. However, in the case of infection with copper-stressed bacteria, though the inoculum consisted of both healthy and injured bacterial cells, there was no visible splenomegaly or liver enlargement (data not shown), suggesting that the copper stress rendered these bacteria incapable of colonizing these organs in the long term and making them susceptible to clearance by the host immune response.

**Figure 3:**
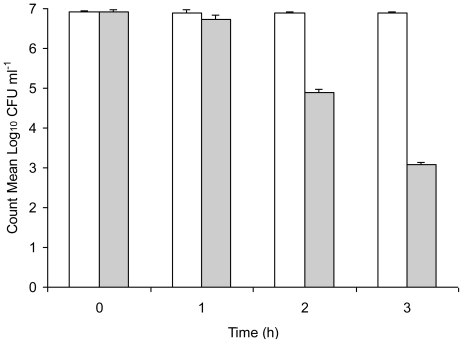
Sensitivity of 2.2 × 10^7^ CFU of unstressed *Salmonella* Typhimurium (unshaded bars) and copper-stressed *Salmonella* Typhimurium (shaded bars) to normal mouse serum. Error bars represent 95% confidence limits (*n* = 3).

Serum is an important host clearance mechanism in infection by pathogenic microbes and we ascertained the relative susceptibility of unstressed *Salmonella* Typhimurium and copper-stressed *Salmonella* Typhimurium to normal mouse serum. Results of the sensitivity of unstressed *Salmonella* Typhimurium and copper-stressed *Salmonella* Typhimurium to normal mouse serum are shown in Figure 3. Though both were able to be enumerated up to 3 h incubation, copper-stressed bacteria demonstrated a gradual decrease of approximately 4 Log_10_ cycles in the count from 0 h to 3 h whereas the count of unstressed *Salmonella* Typhimurium remained consistently high throughout the incubation period, indicating its serum-resistance. Bacteria that were exposed to copper stress were comparatively more susceptible to the antimicrobial action of serum as evidenced by the decrease in colony count with incubation, while unstressed *Salmonella* Typhimurium demonstrated no significant change in bacterial count, thereby demonstrating its maintained resistance to serum and survival in blood for extended time periods.

**Figure 4:**
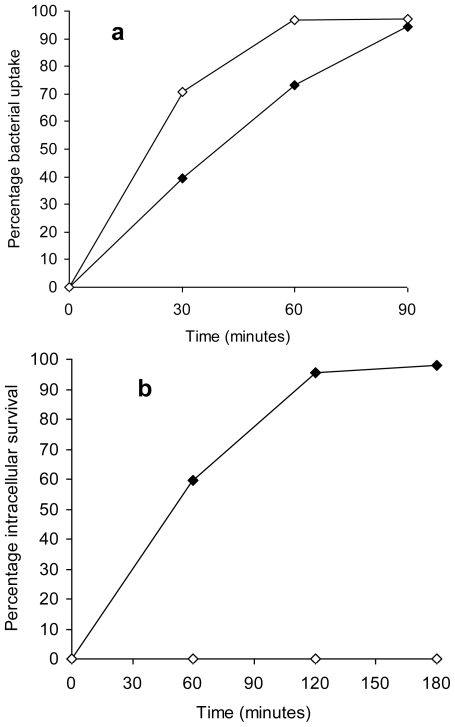
(a) Percentage bacterial uptake of unstressed *Salmonella* Typhimurium (closed symbols) and copper-stressed *Salmonella* Typhimurium (open symbols) by peritoneal macrophages after 0, 30, 60 and 90 min incubation. (b) Percentage intracellular survival of unstressed *Salmonella* Typhimurium (closed symbols) and copper-stressed *Salmonella* Typhimurium (open symbols) within peritoneal macrophages after 0, 60, 120 and 180 min incubation. Error bars represent 95% confidence limits (*n* = 3).

Phagocytic cells are one of the first line of defence against invading micro organisms though previous studies report that *Salmonella* Typhimurium being an intracellular pathogen has mechanisms to evade killing by phagocytes and instead survives within phagocytes of reticuloendothelial system [35]. Not only does the bacteria resist intracellular killing within macrophages, this survival is an essential component of the virulence of *Salmonella* Typhimurium [36]. Both unstressed *Salmonella* Typhimurium and the copper-stressed *Salmonella* Typhimurium demonstrated a gradual increase in bacterial uptake based on counts within peritoneal macrophages from 0 min to 90 min, with the highest percentage uptake following an incubation period of 90 min (Figure 4a). The percentage uptake of healthy cells of the copper-stressed *Salmonella* Typhimurium was higher than for unstressed *Salmonella* Typhimurium at both 30 and 60 min (*p* = 0.002). There was a 100% inactivation of copper-stressed *Salmonella* Typhimurium cells within peritoneal macrophages as indicated by no detectable count on nutrient agar plates under aerobic conditions at all time points. In comparison, approximately 98% survival of unstressed *Salmonella* Typhimurium was observed within peritoneal macrophages at 180 min of incubation (Figure 4b).

We observed a successful uptake of unstressed *Salmonella* Typhimurium by peritoneal macrophages with approximately 98% survival demonstrating the resistance of the bacteria to intracellular killing in accordance with previous studies. In comparison, copper-stressed bacteria, though taken up by macrophages were unable to survive the microbicidal action of phagocytes, as demonstrated by no detectable counts at all time points. Since survival in macrophages is a prerequisite for *Salmonella* infections, this finding indicates that post-copper exposure, *Salmonella* Typhimurium is rendered susceptible to the host immune response and is unable to evade this primary line of defence.

**Figure 5:**
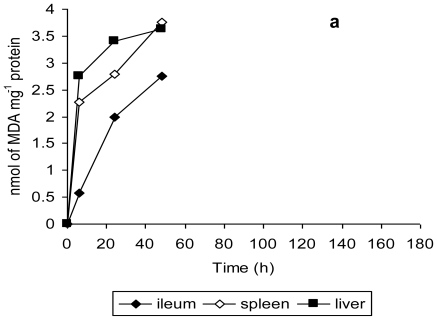

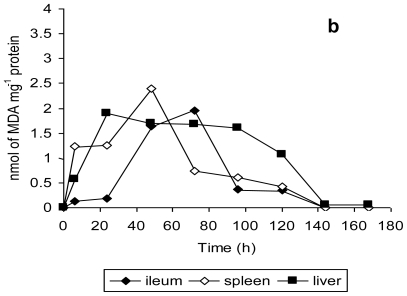
Malondialdehyde in ileum, spleen and liver tissue homogenates of mice orally infected with 2.2 × 10^7^ CFU of (a) unstressed *Salmonella* Typhimurium, and (b) copper-stressed *Salmonella* Typhimurium at 0, 6, 24, 48, 72, 96, 120, 144 and 168 h post infection. Error bars represent 95% confidence limits (*n* = 3). All mice infected with unstressed *Salmonella* Typhimurium died after 48 h.

The findings of this study are further validated by the observations of tissue damage in terms of lipid peroxidation and histopathological studies. Malondialdehyde is one of the most studied secondary product of lipid peroxidation and is quantified as a measure of oxidative stress both *in vitro* and *in vivo* [35]. MDA levels in ileum, spleen and liver tissue homogenates were measured to assess the amount of tissue damage in terms of lipid peroxidation [38]. Mice infected with unstressed *Salmonella* Typhimurium exhibited a gradual increase in MDA level from 6 h to 48 h, the maximum tissue damage being evident at 48 h (Figure 5a). In contrast, the MDA levels in the ileum, spleen and liver tissue homogenates of mice infected with copper-stressed *Salmonella* Typhimurium remained consistently lower at these time points (Figure 5b), compared to those for unstressed *Salmonella* Typhimurium. There was a clear evidence of an increase in tissue damage of ileum and spleen tissue for mice infected with copper-stressed *Salmonella* Typhimurium from 6 h to 72 h followed by a gradual decrease up to 120 h, with no MDA being detected at 144 h and 168 h except in liver tissue, where very low levels of MDA were detectable until 168 h.

**Figure 6:**
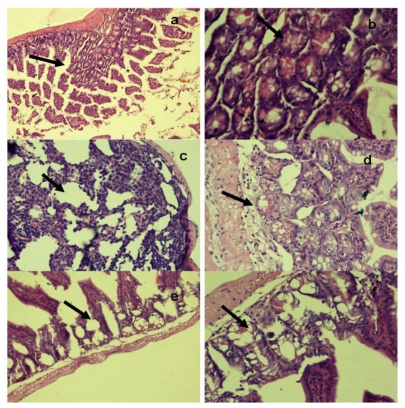
Histopathological examination of ileum tissue of mice infected orally with 2.2 × 10^7^ CFU of unstressed *Salmonella* Typhimurium at (a,b) 6 h, (c,d) 12 h and (e,f) 24 h post-infection.

Histopathological examination of the ileum tissues of mice infected with unstressed *Salmonella* Typhimurium showed increased inter-villus gaps (Figure 6) with mild lymphoid tissue depletion and hyperplasia of Paneth cells at 6 h post-infection. Figure 6a shows increased inter villus gap (H & E × 280) with an increased number, size and density of Paneth cells (Figure 6b) (H & E × 560). By 12 h post-infection, the tips of villi were observed to dissolve, giving a “spire” appearance with loss of nuclei and merging of cytoplasm and basement membranes. There was lympholysis with depletion of lymphocytes (Figure 6c) (H & E × 280) as well as merging of cytoplasm and basement membrane (Figure 6d) (H & E × 560). At 24 h, the villi appeared shrunk and apart (Figure 6e) (H & E × 280) with grossly distended crypts and excess apoptosis of Peyer’s patch (Figure 6f) (H & E × 560). Advanced depletion of ileum mucosa was observed with villi that were shrunk and more widely spaced than in normal tissue. There was also a marked mucosal and crypt dissolution, with substantial apoptosis of lymphoid cells in the lamina propria and Peyer’s patch.

**Figure 7:**
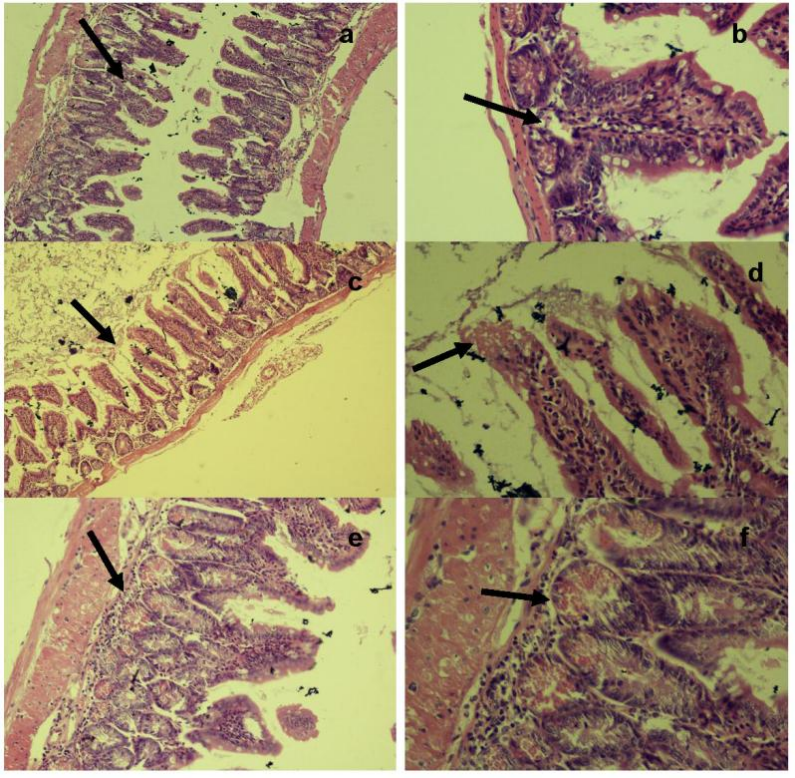
Histopathological examination of ileum tissue of mice infected orally with 2.2 × 10^7^ CFU of copper-stressed *Salmonella* Typhimurium at (a,b) 48 h, (c,d) 72 h and (e,f) 96 h post infection.

In comparison, the ileal villi of mice infected with copper-stressed *Salmonella* Typhimurium exhibited normal architecture and population at 48 h with normal epithelial lining and only mild depletion of goblet cells towards the tip of villi (Figure 7). The ileum showed normal architecture (Figure 7a) (H & E × 280) with normal paneth cells, crypts and submucosa (Figure 7b) (H & E × 560). At 72 h post-infection, there was only mild mucosal lysis with Paneth cell hyperplasia, mucosal villi showed fairly normal body (Figure 7c) (H & E × 280) with slight coagulation at tips and moderate loss of brush border (Figure 7d) (H & E × 560). By 96 h, the mucosa had returned to their normal appearance suggesting possible regeneration of the crypt layer with normal lymphocytes in the lamina propria. The crypt layer appeared thicker (Figure 7e) (H & E × 280) with normal villi and submucosa (Figure 7f) (H & E × 560).

## 4. Conclusions

Copper stress achieved by short term storage (6 h) in a copper water storage vessel conferred a decrease in infectivity of *Salmonella* Typhimurium in normal mice as demonstrated by the decreased pathogenicity in colonizing organs such as ileum, liver and spleen. Though the stressed bacteria were able to circulate systemically by surviving in blood for a short time period, the lytic action of serum coupled with phagocytosis by peritoneal macrophages caused a complete clearance and subsequent recovery from this initial mild infection. Further studies are now required to understand the detailed mechanisms of action of copper stress on individual virulence factors of *Salmonella* Typhimurium in vivo and to develop a more comprehensive understanding of the inactivation phenomenon and its implication in a suitable host.
